# Identity Development and Social-Emotional Disorders During Adolescence and Emerging Adulthood: A Systematic Review and Meta-Analysis

**DOI:** 10.1007/s10964-021-01536-7

**Published:** 2021-11-16

**Authors:** Rachel Potterton, Amelia Austin, Lauren Robinson, Hannah Webb, Karina L. Allen, Ulrike Schmidt

**Affiliations:** 1 Institute of Psychiatry, Psychology and Neuroscience, Section of Eating Disorders, King’s College London, London, United Kingdom; 2 The Eating Disorders Service, Maudsley Hospital, South London and Maudsley NHS Foundation Trust, London, United Kingdom

**Keywords:** Identity development, Mental health, Adolescence, Emerging adulthood, Depression, Anxiety

## Abstract

Depression, anxiety and eating disorders (“social-emotional disorders”) are common during adolescence/emerging adulthood, periods of intense identity development. Despite this, there are few reviews of existing research on the relationship between symptoms of these disorders and ongoing identity development. This study systematically reviewed, narratively synthesized and meta-analyzed longitudinal investigations of the relationship between identity synthesis/confusion and depression, anxiety and eating disorders symptoms during adolescence/emerging adulthood. Three databases (PsycInfo, Medline, Embase) were searched. Study quality was systematically appraised, findings were qualitatively synthesized and (where possible) meta-analyzed. 20 studies (55% “fair” quality, 45% “poor” quality) were identified, including 13,787 participants (54.2% female, mean age = 14.48 years, range 10–29 years). The narrative synthesis found evidence of bidirectional relationships between identity synthesis/confusion and depression, anxiety and eating disorder symptoms. Meta-analyses and meta-regressions of a sub-sample of studies (*N* = 9) indicated no significant associations between identity synthesis or confusion and anxiety or depression symptoms. More high-quality research is needed before firm conclusions can be drawn.

## Introduction

Depression, anxiety and eating disorders (“social-emotional disorders”) are common during adolescence and emerging adulthood (i.e., 10–30 years of age) (Gibb et al., 2010; Kessler et al., 2012). Ongoing identity development may increase vulnerability to social-emotional disorders, whilst such difficulties may also impede identity development (Klimstra & Denissen, 2017). Increased understanding of any link between identity development and social-emotional disorders may help to improve prevention and intervention efforts for these difficulties. However, intervention development remains in its infancy, and rigorous reviews which synthesize existing basic research are needed to advance the field. This study aims to narratively synthesize and meta-analyze the findings of longitudinal studies which have examined prospective associations between identity synthesis and confusion and symptoms of social-emotional disorders during adolescence and emerging adulthood.

Large prospective cohort studies indicate that 40 to 50% of adolescents and emerging adults meet diagnostic criteria for a mental disorder (Gibb et al., 2010; Kessler et al., 2012). Many more young people experience subthreshold symptoms, which can cause distress and impairment and lead to later clinical need (Van Oort et al., 2009). Epidemiological studies indicate that depressive, anxiety and eating disorders are particularly widespread during adolescence and emerging adulthood (Galambos et al., 2006; McLaughlin & King, 2015; Van Oort et al., 2009). Researchers have noted phenotypic similarities between depressive, anxiety and eating disorders (e.g., heightened emotionality and social sensitivity), and suggested that these disorders might be conceptualized collectively as “social-emotional disorders”, and may share etiological and maintenance factors (Rapee et al., 2019).

Developmental psychopathologists suggest that understanding normative adolescent and emerging adult development can provide valuable insights into the mechanisms that eventuate in or maintain social-emotional disorders during these life-stages (Cicchetti & Rogosch, 2002). Self-identity formation is one such normative developmental process for adolescents and emerging adults (Schwartz et al., 2013). During these life-stages, young people seek answers to questions related to who they are and who they would like to be in different life domains (e.g., vocation; relationships; religious beliefs; values) (Schwartz et al., 2013). Whilst several more recent theories of identity development (e.g., narrative identity; McAdams, 2011) have been proposed, Erik Erikson’s seminal theory remains the dominant theoretical framework (Schwartz et al., 2013). It proposes that successful identity development involves moving from a state of predominant “identity confusion” (i.e., a sense of inconsistency or uncertainty in one’s idea of oneself) towards “identity synthesis” (i.e., a sense of sameness and continuity of the self; Erikson, 1950). “Exploration” (i.e., trying various identity options) and “commitment” (i.e., selecting identity options) were later proposed as the key processes underpinning identity confusion and synthesis (Marcia, 1966). Based on levels of past and current exploration and commitment, individuals can be categorized into four discrete identity statuses: “diffusion”; “moratorium”; “foreclosure” and “achievement” (Marcia, 1966). Subsequent neo-Eriksonian models retain a focus on the interplay between synthesis and confusion, with variations in how exploration and commitment are conceptualized (Crocetti et al., 2008; Luyckx et al., 2008a) (see Table 1 for summary of these processes). Several questionnaires have been developed to measure these processes (e.g., Dimensions of Identity Development Scale (DIDS); Utrecht Management of Identity Commitments Scale (UMICS); Crocetti et al., 2010; Luyckx et al., 2008a).

**Table 1:** Summary of identity confusion and synthesis-related concepts

Concept	Definition
*Identity synthesis*	
Commitment-making	Committing to a set of identities
Identification with commitment	Embracing one’s identity commitments and integrating them into the sense of self
Exploration in depth	Exploring existing identity commitments
*Identity confusion*	
Reconsideration of commitment	Comparing existing identity commitments to other possible options
Exploration in breadth	Exploring identity options prior to commitment
Ruminative exploration	Ruminating over identity options

There has been longstanding interest in the connection between mental health and identity synthesis/confusion during adolescence and emerging adulthood (Klimstra & Denissen, 2017). A number of longitudinal and cross-sectional studies have identified that identity confusion is positively correlated with depressive and anxiety disorder symptoms (Crocetti et al., 2011; Luyckx et al., 2015; Luyckx et al., 2013a; Luyckx et al., 2008a; Marcotte and Levesque, 2018; Sica et al., 2014). Researchers have suggested that the relationship between identity confusion/synthesis and social-emotional disorders may operate in a similar way to the relationship between personality traits (e.g., perfectionism, neuroticism) and depression and anxiety symptoms. Several hypotheses have been outlined (see Table 2 for a summary); in essence, identity development difficulties may increase the likelihood of the emergence of social-emotional disorders, social-emotional disorders may increase the likelihood of identity development difficulties, or both phenomena may be underpinned by common causes (Klimstra & Denissen, 2017).

**Table 2:** Putative hypotheses regarding the relationship between identity synthesis/confusion and social-emotional disorders (based on Klimstra & Denissen, 2017)

1.	Identity development difficulties (e.g., high identity confusion/low identity synthesis) and social-emotional disorders have common causes
2.	High identity confusion/low identity synthesis and social-emotional disorders form a continuous spectrum
3.	High identity confusion/low identity synthesis are precursors of social-emotional disorders
4.	High identity confusion/low identity synthesis predispose to developing social-emotional disorders
5.	High identity confusion/low identity synthesis have patho-plastic effects on social-emotional disorders
6.	High identity confusion/low identity synthesis are state-dependent concomitants of social-emotional disorders
7.	High identity confusion/low identity synthesis are consequences of social-emotional disorders

Increased understanding of any link between identity synthesis and confusion and social-emotional disorders in young people may help to improve prevention and intervention efforts for these difficulties. Indeed, some identity-focused interventions do already exist. For example, the Miami Identity Development Project is a peer-led intervention aims to promote identity synthesis through in-depth exploration and working through identity-related distress (Meca et al., 2014). However, intervention development remains in its infancy, and rigorous reviews which synthesize existing basic research are needed to advance the field.

## Current Study

This article aims to narratively synthesize and meta-analyze the findings of longitudinal studies which have examined prospective associations between identity synthesis and confusion and symptoms of social-emotional disorders during adolescence and emerging adulthood (10–30 years). It is hypothesized that identity will positively predict, and identity synthesis negatively predict symptoms of social-emotional disorders in adolescents and emerging adults (Hypothesis 1). Additionally, it is hypothesized that symptoms of social-emotional disorders will positively predict identity confusion and negatively predict identity synthesis in adolescents and emerging adults (Hypothesis 2).

## Methods

A systematic review was conducted in accordance with the relevant PRISMA statement guidelines (Moher et al., 2009). Findings of all studies were narratively synthesized. Studies with relevant data were meta-analyzed and meta-regressions were conducted.

### Search Strategy

Eriksonian and neo-Eriksonian identity development-related search terms were decided based on relevant reviews (Schwartz, 2001; Schwartz et al., 2013; Schwartz et al., 2009), and augmented by key words from preidentified relevant arcticles (see Appendix for full search strategy). Social-emotional disorder-related search terms were designed to capture social-emotional disorders which tend to have their onset during adolescence and emerging adulthood (i.e., depressive, anxiety, eating and psychotic disorders)(Rapee et al., 2019). Three electronic databases (Medline, Embase, and PsycInfo) were searched from inception until 22^nd^ June 2021. Database searches were supplemented by internet searches and hand-searches of reference lists of potentially relevant articles.

### Selection Process

Prior to study selection, eligibility criteria were specified (see Table 3). Studies focused exclusively on ethnic identity development were excluded because its development is conceptualized distinctly from other forms form of identity (Phinney & Ong, 2007). Given limited resources—and as is widespread in systematic reviews—non-English studies and gray literature (i.e., research unpublished or published in noncommercial form) were also excluded from the review (Hartling et al., 2017). Titles and abstracts of all retrieved publications were imported into EndNote and duplicates were removed by one reviewer (RP). Two reviewers (RP, AA) independently screened the titles and abstracts of retrieved articles. Articles that were deemed highly unlikely to be relevant based on their title and abstract were disregarded. Full text versions of the remaining articles were then obtained and screened independently by the same reviewers. All articles that did not meet the inclusion criteria were excluded, and reasons for their exclusion documented. Discrepancies between the reviewers’ decisions were resolved through discussion. No third-party was consulted.

**Table 3:** Study inclusion and exclusion criteria

Characteristic	Included	Excluded
*Report characteristics*		
Publication type	Peer-reviewed journal articles	Book chapters Conference abstracts Unpublished dissertations
Language	English	Any other language
Accessibility	Full text available online	Full text not available online
*Study characteristics*		
Design	Longitudinal	Cross-sectiona**l**
Methodology	Quantitative	Qualitative Review
Measure of identity development	(Neo)Eriksonian measures of identity development (e.g. DIDS, EPSI)	No (Neo)Eriksonian measure of identity development Ethnic identity focused measures
Measure of social-emotional disorders	Measures of social-emotional disorders (i.e., depression, anxiety, eating disorder, psychosis symptoms)	No measure of social-emotional disorders
Sample	At least 95% of sample adolescents and/or emerging adults (i.e., individuals aged between 10 and 30 years)	5% or more of the sample adults (i.e., individuals aged >30 years)

Abbreviations: DIDS dimensions of identity development scale, EPSI erikson psychosocial stage inventory

### Data Extraction

One reviewer (RP) extracted data concerning sample characteristics, measures and main findings from the included articles using a prepiloted data form. These data were independently checked (i.e., extracted data were compared to those reported in the included articles) by a second reviewer (AA). All discrepancies were resolved through discussion.

### Quality Assessment

The quality of included studies was independently appraised by two reviewers (RP and AA) using the Newcastle Ottawa Quality Assessment Scale (NOS)(Wells et al., 2014). This scale was developed to assess the quality of nonrandomized studies in systematic reviews and meta-analyses and is recommended for use by the Cochrane Collaboration (Higgins et al., 2019). The NOS judges study quality on eight items from three broad criteria: (i) the selection of the study groups; (ii) comparability of the groups e.g., by controlling for relevant baseline factors, and (iii) the ascertainment of the outcome of interest. A maximum score of nine can be given to a study and thresholds for converting the NOS to Agency for Healthcare Research and Quality standards are as follows: “Poor”: selection ≤1 OR comparability = 0 OR outcome/exposure ≤1; “Fair”: selection = 2 AND comparability ≤2 AND outcome/exposure ≤3; “Good”: selection ≤4 AND comparability ≤2 AND outcome/exposure ≤3. The scale’s inter–rater reliability has been found to range from fair to high depending on the types of studies assessed and the raters (Hartling et al., 2013; Oremus et al., 2012). Other psychometric properties of the scale have not been published. Ratings were compared between reviewers and consensus achieved through discussion.

### Narrative Synthesis

All studies were narratively synthesized (i.e., summarized and explained using words and text). In line with relevant guidance, findings were first organized to describe patterns across the studies in terms of significant effects, their size and direction, before factors which might explain any differences across the included studies were considered (Popay et al., 2006). The rationale for using narrative synthesis was two-fold. Firstly, narrative synthesis aided the decision as to what other synthesis methods were appropriate. It was also anticipated that due to study heterogeneity, it would not be possible to include all studies in the meta-analyses. Although narrative synthesis is a less rigorous and reliable method of synthesizing evidence than meta-analytic techniques, it is a credible alternative when heterogeneity is high and not all studies can be included in meta-analyses (Popay et al., 2006).

### Meta-analyses and Meta-regression

#### Study selection and data extraction

Meta-analyses were considered for each dependent variable of interest (i.e., identity synthesis, identity confusion, depression, anxiety, eating disorder symptoms). Studies were considered for inclusion in the meta-analyses if they reported relevant data (i.e., means and standard deviations of the dependent variable at two or more time-points). Where studies reported multiple follow-up time-points, data pertaining to the last time-point were extracted. Efforts were made to ensure that duplicate studies were not included. Where studies were deemed to be exact duplicates (i.e., same cohort and same sample size) or near duplicates (i.e., same cohort and ≤ 50% unique participants), only the study with the longest follow-up period was included. Where studies appeared to report on overlapping but not exact or near duplicate samples (i.e., same cohort but ≥50% unique participants), all studies were included. This strategy was deemed appropriate given the relatively small number of studies identified by the searches, and the need to maximize the amount of data included in the meta-analyses.

Based on the data available, the aims of the review and the results of the narrative synthesis, it was decided to conduct three meta-analyses: change in (i) identity synthesis, (ii) identity confusion and (iii) depression and anxiety symptoms over adolescence/emerging adulthood. As only two studies reported data on eating disorder symptoms, it was not possible to conduct a meta-analysis of change in eating disorder symptoms due to insufficient data. Just two studies reporting the required data used a measure of anxiety only, and several studies assessed anxiety and depression as a composite. For this reason, change in depression and anxiety symptoms were examined together, rather than in separate meta-analyses. When studies reported on more than one aspect of the identity variables (e.g., measured identification with commitment and commitment making, both conceptually tied to identity synthesis), means and standard deviations were combined to create a composite score. Effect sizes (Cohen’s d) for the change in variables of interest between baseline and follow-up were calculated using the mean scores at follow-up minus mean scores at baseline, divided by the pooled pretest standard deviation (i.e., standardized mean difference) (Dunning et al., 2019).

Relevant data were also extracted from the included studies for the meta-regression analyses. Alongside predictors of putative relevance (e.g., age at baseline, follow-up duration), six continuous predictors were defined based on the available data and the aims of this review: (i) change in depression and anxiety symptoms; (ii) baseline depression symptoms; (iii) change in identity synthesis; (iv) change in identity confusion; (v) baseline identity synthesis; (vi) baseline identity confusion. Data on change in depression and anxiety symptoms and identity synthesis and confusion over time (i.e., standardized mean difference—mean scores at follow-up minus mean scores at baseline, divided by the pooled pretest standard deviation) were available for all studies. As sufficient data were not available to standardize baseline identity and depression/anxiety scores, only studies who provided raw baseline scores on the same continuous measure (i.e., the same scale) were able to be included in these continuous predictors. Data were extracted from studies (*N* = 4) which utilized the most used questionnaire (the CES-D), a measure of depression symptoms. Similarly, data on baseline identity synthesis and confusion were derived from studies (*N* = 4) which utilized the most used measure (the UMICS) only.

#### Data analysis

Due to heterogeneity of studies’ measures and analytic techniques, a stepwise approach was used. Meta-analyses were first conducted to assess whether there were any significant changes in depression and anxiety symptoms, identity synthesis and identity confusion over time. Meta-regression analyses were then conducted to investigate the effect of key variables of interest – including baseline and change scores for identity synthesis and confusion and depression and anxiety symptoms – on each statistically significant meta-analysis outcome.

All meta-analyses and meta-regressions were conducted in Stata/SE 16.0 (StataCorp, L., 2019) using the “meta” command. The standardized mean difference (SMD) was used in the summary statistics, which expresses the size of the effect in each study (baseline vs. follow-up), relative to the variability observed in each study. For all meta-analyses, a random effects model was specified using the DerSimonian and Laird method (DerSimonian & Laird, 1986). This was chosen because heterogeneity was high. The random effects model assumes both within-group variability and between-study heterogeneity. Heterogeneity between study findings was assessed by calculating Higgins I^2^ (Higgins et al., 2003) based on Cochran’s Q indexes. I^2^ measures the percentage of total variation across studies due to heterogeneity. Publication bias was assessed using the Duval and Tweedie trim and fill method (Duval & Tweedie, 2000), which identifies and adjusts for funnel plot asymmetry, and Eggers test for small study effects (Egger et al., 1997).

## Results

### Study Selection and Characteristics

**Fig. 1:**
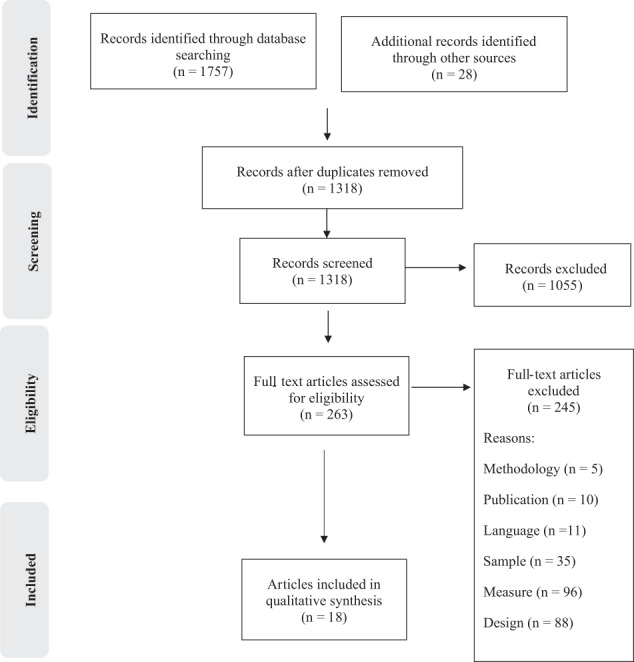
PRISMA flow chart of study screening and selection

The systematic search yielded a total of 1318 records following deduplication. After screening of abstracts and closer examination of full text arcticles by both reviewers, 1300 articles were excluded as not relevant (see Fig. 1). Consensus between the reviewers following independent screening of full texts was 88%. The review therefore included a total of 18 articles, reporting 20 studies.

### Characteristics of Included Studies

Details concerning study sample size and characteristics, measures, and main findings of the included studies are summarized in Table 1, Supplementary Materials/Online Resource 1. Studies include a total of 13,787 participants, and all were nonclinical, community-based samples. 12 studies (60.0%) included adolescent participants only (range: 10–18 years), one study (5.0%) emerging adults only (range: 17–29 years), and seven studies (35.0%) included both adolescents and emerging adult participants (range: 10–24 years). Three studies (15.0%) focused on eating disorder symptoms; the remainder measured depression and anxiety symptoms. All studies used self-report measures of both identity development and mental health problems. No included studies assessed the efficacy of identity-focused clinical interventions.

### Quality Assessment

Consensus between the reviewers following independent rating of study quality was 86%. After discussion, consensus was 100% and the majority (*N* = 11, 55%) of the studies were judged to be of “fair” quality (i.e., selection of the study groups = 2 AND comparability of the groups ≤2 AND ascertainment of outcome/exposure ≤3). The remaining studies were all judged to be of “poor” quality (i.e., selection of the study groups ≤1 OR comparability of the groups = 0 OR ascertainment of outcome/exposure ≤1), with no studies rated as “good” quality (i.e., selection of the study groups ≤4 AND comparability of the groups ≤2 AND ascertainment of outcome/exposure ≤3). Quality ratings for each included study can be found in Table 1, Supplementary Materials/Online Resource 1. All studies (poor and good quality alike) were considered for further analyses.

### Narrative Synthesis

#### Eating disorder symptoms

Of the three studies exploring eating disorder symptoms, one study categorized adolescents into groups according to their eating disorder symptoms and body mass index (BMI) over time (Verschueren et al. 2019). This study found that a “high risk” eating disorder symptom trajectory (i.e., normal BMI + high levels of eating disorder symptoms) had higher levels of identity confusion compared to “low risk” groups (i.e., normal/low BMI + low levels of eating disorder symptoms).

Another study by Verschueren and colleagues assessed whether eating disorder symptoms predicted identity development later in adolescence, and vice versa (Verschueren et al., 2018). They found that high body dissatisfaction and bulimia symptoms (but not drive for thinness) predicted lower identity synthesis and higher identity confusion one and two years later amongst Dutch adolescents, with small effect sizes. High identity synthesis also predicted low eating disorders symptoms (body dissatisfaction; bulimia; drive for thinness) and high identity confusion predicted high body dissatisfaction and bulimia one and two years later, with small effect sizes. There was no prospective association between identity confusion and drive for thinness. Finally, a recent study by Verschueren and colleagues in Belgian adolescents and emerging adults found that identity synthesis and confusion predicted eating disorder symptoms one and two years later (Verschueren et al., 2021).

### Depression and anxiety symptoms

Of the 17 studies exploring depression and anxiety symptoms, five focused on classifying individuals into distinct identity development and / or anxiety and depression trajectory classes and investigating overlap between these trajectories (Becht et al., 2016; Hatano et al., 2019; Luyckx et al., 2008b; Meeus et al., 2012). Several found that adolescents and emerging adults in identity development trajectories characterized by high synthesis and low confusion (i.e., achievement; foreclosure) had fewer depression and anxiety symptoms than those in low synthesis and high confusion trajectories (i.e., moratorium; diffusion) (Becht et al., 2016; Hatano et al., 2019; Luyckx et al., 2008b; Meeus et al., 2012). There was also some evidence that high anxiety trajectories were characterized by low synthesis and high confusion in adolescents, although one study found that this pertained to school-related anxiety symptoms only, and not social and generalized anxiety (Crocetti et al., 2009; Nelemans et al., 2014).

Eleven studies (in ten publications) focused on associations between identity development and depression and anxiety symptoms (Becht et al., 2019; Hatano et al., 2020; Meca et al., 2019; Meca et al., 2017; Schwartz et al., 2012; Schwartz et al., 2011; Schwartz et al., 2017; van Doeselaar et al., 2018). Of the studies which assessed the impact of depression and anxiety symptoms on later identity development, two studies found that depression and anxiety symptoms did not significantly predict later identity synthesis in Japanese and Dutch adolescents (Hatano et al., 2020; van Doeselaar et al., 2018). However, one of these studies found that high depression and anxiety symptoms did predict higher identity confusion (Hatano et al., 2020). Another such study found that high depressive symptoms predicted lower educational (but not interpersonal) identity synthesis four years later, with a small effect size (van Doeselaar et al., 2018). There was also evidence from one study for a differential effect of anxiety and depressive symptoms, such that high depression—but not anxiety—predicted lower later identity synthesis in adolescents (Schwartz et al., 2012). Interestingly the same study found that high anxiety—but not depression—predicted higher identity confusion one year later, with a small effect size. Three studies (reported in two publications) found no evidence of a prospective association between within-person changes in depression and anxiety symptoms and later identity synthesis and confusion in adolescents and emerging adults (Becht et al., 2019; Hatano et al., 2020). However, another study did report such an effect—within-person increases in depressive symptoms were associated with decreases in identity synthesis and increases in identity confusion six months later during adolescence, with small effect sizes (Meca et al., 2019).

Regarding effects in the opposite direction (i.e., impact of identity development on later depression and anxiety symptoms), four studies (in three samples) found no prospective association between identity synthesis and later depression and anxiety symptoms in adolescents (Hatano et al., 2020; Meca et al., 2017; Schwartz et al., 2011; Schwartz et al., 2017). These studies’ findings were more mixed in relation to identity confusion; two found that elevated identity confusion predicted higher depressive symptoms with small effect sizes (Meca et al., 2017; Schwartz et al., 2017), but one found no such effect (Hatano et al., 2020). Another study found that high interpersonal (but not educational) identity synthesis predicted lower depressive symptoms three years later in adolescents and emerging adults, although this finding was not replicated in another study in the same publication (van Doeselaar et al., 2018). One further study found evidence of a differential effect of anxiety and depressive symptoms—high identity synthesis predicted lower depressive symptoms in adolescents but found no such effect for anxiety disorder symptoms (Schwartz et al., 2012). Another study found that experiencing fluctuations (i.e., day to day change over a three-week period) in identity synthesis did not predict depression and anxiety symptoms one year later during adolescence, but fluctuations in identity confusion did, with a small effect size (Schwartz et al., 2011). Three studies (reported in two publications) found no evidence for an effect of within-person change in identity synthesis but did find such an effect for identity confusion in adolescents and emerging adults (Becht et al., 2019; Hatano et al., 2020). Another study found the opposite pattern of results; within-person increases in identity synthesis were associated with decreases in depressive symptoms six months later – with a small effect size – during adolescence, but there was no effect of identity confusion (Meca et al., 2019).

### Meta-Analyses

**Fig. 2:**
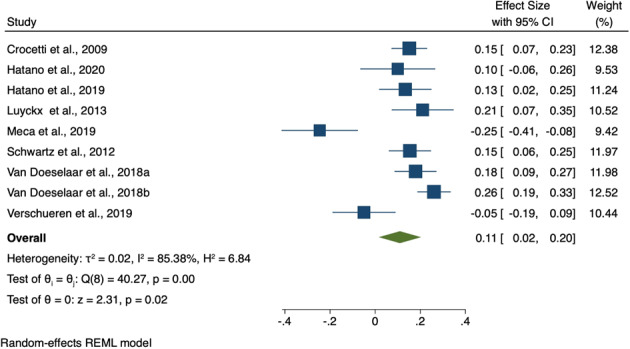
Forest plot of effect sizes in meta-analysis 1 (i.e., Change in identity synthesis over adolescence/emerging adulthood)

**Fig. 3:**
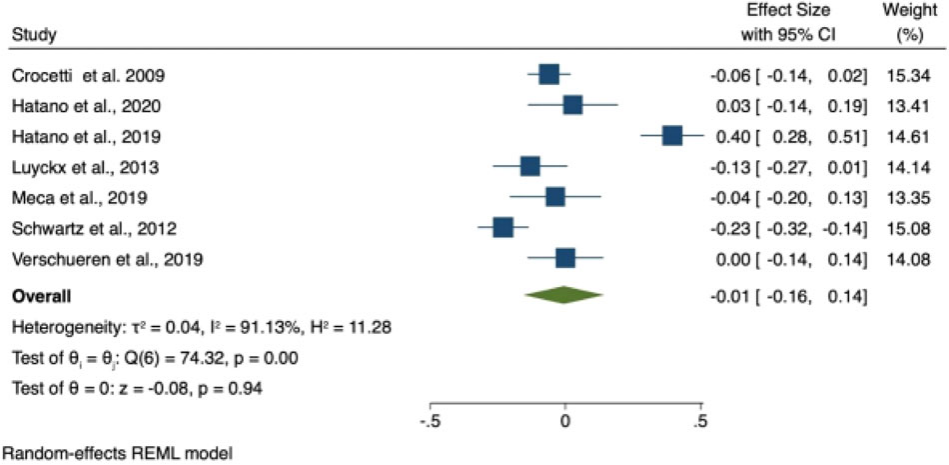
Forest plot of effect sizes in meta-analysis 2 (i.e., Change in identity confusion over adolescence/emerging adulthood)

Three meta-analyses were conducted: change in (i) identity synthesis, (ii) identity confusion and (iii) depression and anxiety symptoms over adolescence/emerging adulthood. Nine studies had data available to be included in at least one of the three meta-analyses. Details concerning study sample size and characteristics are summarized in Table 4; extracted data are presented in Table 4, Supplementary Materials/Online Resource 1; reasons for exclusion are presented in Table 1, Supplementary Materials/Online Resource 1. Results for all three meta-analyses are summarized in Table 5 and in forest plots seen in Figs. 1–3.

**Table 4:** Characteristics of studies included in the meta-analyses

Authors / Cohort Acronym	Sample	*N*	Age	Follow-up	Measures	Quality	Meta-analysis
Crocetti et al. (2009) CONAMORE	53% female Ethnicity NR	1313	12.42 ± 0.59	5 years	UMICS SCARED	Fair	1, 2, 3
Hatano et al. (2020) JLIRP	53% female Ethnicity NR	347	14 ± NR	3 years	DIDS SDQ	Fair	1, 2, 3
Hatano et al. (2019) JLIRP	50% female Japanese	968	NR 13–16 yrs.	4 years	UMICS SDQ	Poor	1, 2, 3
Luyckx et al. (2013a) Unspecified Cohort	85% female uni students 94% Caucasian	456	18.3 ± 1.4	9 months	DIDS ISRI CES-D	Poor	1, 2, 3
Meca et al. (2019) COPAL	47% female Hispanic recent migrants	302	14.5 ± 0.9	3 years	EPSI CES-D	Fair	1, 2, 3
Schwartz et al. (2012) (Unspecified Cohort)	49% female Ethnicity NR	923	12.4 ± 0.6	5 years	UMICS SCARED CDI	Poor	1, 2, 3
van Doeselaar et al. (2018) a CONAMORE	57% female 92% Dutch	951	13.6 ± 2.0	8 years	UMICS CDI	Fair	1, 3
van Doeselaar et al. (2018) b USAD	57% female	1904	18.3 ± 3.8	3 years	UGIDS GHQ	Fair	1, 3
Verschueren et al. (2019) (Unspecified Cohort)	51% female Ethnicity NR	1528	15.0 ± 1.8	3 years	EPSI EDI-3 HADS	Poor	1, 2, 3

Abbreviations: CDI Children’s Depression Inventory, CES-D Center for Epidemiologic Studies Depression Scale, DIDS Dimensions of Identity Development Scale, EDI-3 Eating Disorder Inventory Version 3, EPSI Erikson Psychosocial Stage Inventory, GHQ General Health Questionnaire, HADS Hamilton Anxiety and Depression Scale, ISRI Identity Stage Resolution Index, NR Not Reported, SCARED Screen for Child Anxiety Related Disorders, SD Standard Deviation, SDQ Strengths and Difficulties Questionnaire, UGIDS Utrecht Groningen Identity Development Scale, U-MICS Utrecht Management of Identity Commitments Scale

**Table 5:** Meta-analyses 1, 2 and 3: Summary of comparative outcomes

	*N* (Baseline, follow-up)	Pooled effect size	L 95% CI	U 95% CI	Z	*P*
Identity synthesis	7682, 6000	0.11	0.02	0.20	2.31	0.02
Identity confusion	4930, 3807	−0.01	−0.16	0.14	−0.08	0.94
Depression and anxiety symptoms	7751, 6054	−0.11	−0.18	−0.03	−2.84	0.00

Abbreviations: *N* sample size, *P* p value, L lower, U upper

#### Identity synthesis

Nine studies provided data on change in identity synthesis over adolescence/emerging adulthood and were included in this meta-analysis. These studies included a total of 8692 participants. Seven studies (77.8%) included adolescent participants only, one study (11.1%) emerging adults only, and one study (11.1%) included both adolescents and emerging adult participants. Five studies (55.6%) were rated as fair quality and the remainder as poor quality. The meta-analysis found a significant increase in identity synthesis over time (SMD = 0.11; 95% CI 0.02–0.20; *p* = 0.02), Fig. 2.

#### Identity confusion

Seven studies provided data on changes in identity confusion over adolescence/emerging adulthood and were included in the meta-analysis. These studies included a total of 5837 participants. Six studies (66.7%) included adolescent participants only and one study (14.3%) emerging adults only. Four studies (57.1%) were rated as poor quality, and the remainder as fair quality. The meta-analysis found no significant change in identity confusion over time (SMD = −0.01; 95% CI −0.16 to 0.14; *p* = 0.94), Fig. 3.

#### Depression and anxiety symptoms

**Fig. 4:**
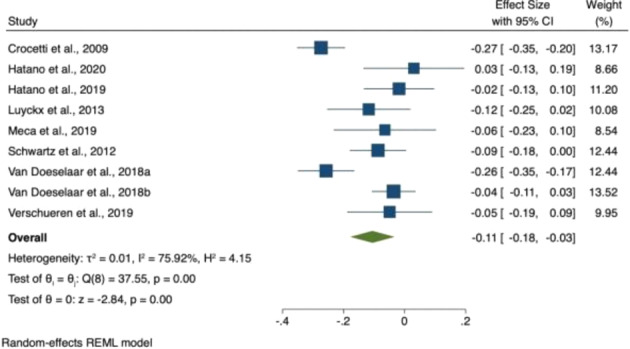
Forest plot of effect sizes in meta-analysis 3 (i.e., change in depression and anxiety symptoms over adolescence/emerging adulthood)

Nine studies provided data on changes in depression and anxiety symptoms over adolescence/emerging adulthood and were included in the meta-analysis. These studies included a total of 8692 participants. Seven studies (77.8%) included adolescent participants only, one study (11.1%) emerging adults only, and one study (11.1%) included both adolescents and emerging adult participants. Five studies (55.6%) were rated as fair quality, and the remainder as poor quality The meta-analysis found a significant decrease in depression and anxiety symptoms over time (SMD = −0.11; 95% CI −0.18 to −0.03; *p* = 0.005) Fig. 4.

### Meta-regression

Following the significant changes between baseline and follow-up measures of both identity synthesis and depression and anxiety symptoms among the included studies, meta-regression analyses were performed to identify key predictors of change. The effect of age at baseline, follow-up duration, and baseline and change scores for depression and anxiety symptoms were all investigated as predictors of change in identity synthesis, with no statistically significant predictors identified. In addition, the effect of age at baseline, follow-up duration, and baseline and change scores for identity synthesis and identity confusion were investigated as predictors of change in depression and anxiety symptoms, with no statistically significant predictors identified. Results are presented in Table 6.

**Table 6:** Meta-regression results

Covariate	Coefficient	L 95% CI	U 95% CI	*P*
Meta-regression #1: Identity synthesis				
Baseline depression symptoms	−0.15	−2.23	1.92	0.78
Change in depression and anxiety symptoms	−0.31	−1.53	0.92	0.57
Study duration	0.11	−0.05	0.08	0.69
Baseline age	0.02	−0.04	.07	0.52
Meta-regression #2: Depression and anxiety symptoms				
Baseline identity synthesis	−0.37	−1.54	0.80	0.31
Change in identity synthesis	−4.69	−19.83	10.46	0.32
Baseline identity confusion	−0.09	−4.80	4.62	0.84
Change in identity confusion	0.19	−0.46	0.84	0.50
Study duration	−0.03	−0.07	0.00	0.08
Baseline age	0.02	−0.02	0.06	0.29

### Sensitivity Analysis

The Higgins I^2^ heterogeneity statistic ranged from 75.92% to 91.13% across our three sets of analyses, justifying the use of the random-effects model. The Egger test indicated small study effects within the identity synthesis meta-analysis (*t* = −5.61, *p* = 0.00), but not in the identity confusion (*t* = 1.50, *p* = 0.77) and depression and anxiety symptoms (*t* = 2.93, *p* = 0.16) meta-analyses. The trim and fill correction was performed and pooled effect sizes were converted to an exponential form, with key findings for change in identity synthesis (SMD = 0.11; 95% CI: 0.01–0.203) and depression and anxiety symptoms (SMD = −0.132; 95% CI: −0.201 to −0.06) remaining statistically significant.

## Discussion

Social-emotional disorders are common during adolescence/emerging adulthood, times of intense identity development. Increased understanding of any link between identity development and social-emotional disorders in young people may help to improve prevention and intervention efforts for these difficulties, yet few reviews synthesize existing basic research exploring this relationship. This study aimed to narratively synthesize and meta-analyze the findings of longitudinal studies which have examined prospective associations between identity synthesis and confusion and symptoms of social-emotional disorders during adolescence and emerging adulthood.

### Narrative Synthesis

The narrative synthesis found partial support for the hypotheses of this review. Several studies found evidence for an overlap between identity development and depression and anxiety symptoms, such that adolescents and emerging adults in identity development trajectories characterized by high synthesis and low confusion had fewer depression and anxiety symptoms than those in identity development trajectories characterized by low synthesis and high confusion (Becht et al., 2016; Crocetti et al., 2009; Hatano et al., 2020; Luyckx et al., 2008b; Meeus et al., 2012; Nelemans et al., 2014).

There was some evidence that depression, anxiety and eating disorder symptoms impact identity development, such that elevated symptoms were associated with identity development difficulties (i.e., low identity synthesis/high identity confusion) later in adolescence (Hatano et al., 2020; Schwartz et al., 2012; van Doeselaar et al., 2018; Verschueren et al., 2018; Verschueren et al., 2021). Preliminary evidence indicated that effects may differ according to identity domain (i.e., depressive symptoms associated with later educational synthesis, but not interpersonal synthesis) (van Doeselaar et al., 2018).

Evidence regarding effects in the opposite direction (i.e., impact of identity development on later depression and anxiety symptoms) was mixed. Some studies indicated that difficulties with identity development were associated with more depressive symptoms later in adolescence, but others found no effect (Becht et al., 2019; Hatano et al., 2020; Hatano et al., 2018; Meca et al., 2017; Schwartz et al., 2012; Schwartz et al., 2011; Schwartz et al., 2017).

The narrative review therefore found some support for bidirectional relationships between identity confusion/synthesis and social-emotional disorders i.e., that high identity confusion and low identity synthesis are both a cause and a consequence of social-emotional disorders. However, it is important to note that many studies did not control for identity development/social-emotional disorder at baseline so it is difficult to be certain about the direction of the effect. Furthermore, it is possible that both identity confusion and social-emotional disorders can be explained by a common factor not assessed or controlled for within these studies. Indeed, both social-emotional disorder and identity development difficulties might be better explained by other normative developmental processes (e.g., cognitive development, attachment).

### Meta-analyses

The meta-analyses did not support the hypotheses of this review. Contrary to expectation, there was no significant change in identity confusion over time and a meta-regression was therefore not conducted. Visual inspection of the forest plot suggests that two studies - by the same research group, and drawing participants from the same cohort - found effects in the opposite direction to the other studies (i.e., increasing identity confusion), driving this counter-intuitive finding (Hatano et al., 2019; Hatano et al., 2020). The most apparent difference between these studies and the others is that participants were young people in Japan; it may be that cross-cultural differences in identity development could explain the disparity in findings. The meta-analyses did find a decrease in anxiety and depression over adolescence/emerging adulthood, but this decrease was not significantly predicted by the identity variables (baseline and change in identity synthesis and confusion). Likewise, identity synthesis increased over adolescence/emerging adulthood, but such change was not significantly predicted by either change in depression and anxiety symptoms or baseline depression.

There was therefore a discrepancy between the findings of the narrative synthesis and that of the meta-analyses. As the meta-analyses included a sub-sample of the studies included in the narrative synthesis, differences in included and excluded studies may explain these disparate findings. However, included and excluded studies appear broadly similar in terms of age-group, quality rating and other extracted characteristics. It is also possible that, as only nine studies were included in the meta-analyses, the meta-regressions may not have been sufficiently powered to detect an effect (Higgins et al., 2019).

### Limitations

This study’s findings must be interpreted in light of some limitations. Gray literature and non-English studies were excluded from the review. Whilst previous studies have highlighted that non-English and gray literature studies represent only a very small percentage of the total studies included in reviews and are associated with negligible change in results (Hartling et al., 2017), it is possible that potentially informative studies were not identified which may bias the findings. Studies were predominantly conducted in the Netherlands and the United States, which may limit the generalizability of the review’s findings. There are likely to be differences between these countries and other countries regarding identity development and its association with mental health. Furthermore, most of the studies included in this review were only of fair quality. Studies demonstrated shortfalls in selection and comparability of study groups (e.g., nonrepresentative samples; relevant factors not controlled for at baseline) and measurement of outcomes (e.g., self-report questionnaires; <70% retention rates). Finally, the meta-analyses were also subject to some limitations. It was not possible to include all identified studies in the meta-analyses due to heterogeneity of sample design. Additionally, the meta-analyses included multiple publications drawing on participants from the same cohort. It is possible that this may bias the estimates of effect size.

### Directions for Future Research

The present review highlights several gaps in the existing evidence base, which might be usefully addressed in future research. Future studies should aim to address the quality deficits of existing research, for instance by recruiting representative samples, controlling for relevant factors, and maximizing participant retention across follow-up phases. As identified, most existing research has focused on depression and anxiety. Whilst these types of difficulties are of obvious relevance to adolescent and emerging adult populations, it important to also investigate other disorders (e.g., eating disorders) that are also prevalent and are associated with significant impairment amongst young people. Additionally, all studies included in this review adopted a dimensional approach to depression, anxiety and eating disorders. Future studies might usefully also assess identity development in young people who meet criteria for clinical diagnoses, a more rigorous test of clinically meaningful symptom change.

Most existing research has been conducted in adolescents, yet it is likely that associations between identity development and depression, anxiety and eating disorders symptoms vary according to age. Indeed, previous cross-sectional research has highlighted age graded associations between identity process and mental health problems (e.g., Luyckx et al., 2013a; Luyckx et al., 2008a, 2008b). Further research should focus on elucidating prospective associations between identity development and social-emotional disorders amongst emerging adults, whilst ideally including age-based comparison groups.

Most of the studies included in this systematic review examined non-domain-specific identity development, or interpersonal and educational domains. There is growing evidence that young people who belong to sexual orientation minorities and with gender identity concerns face significant mental health challenges, yet there is an evident lack of studies researching how development of sexual and gender identity is related to mental health. Furthermore, few of the studies assessed other factors that may influence associations between identity development and depression, anxiety and eating disorders. Many factors, both proximal and distal, are likely to influence this relationship (e.g., family, school and peer context; childhood trauma; genetic influences) (Klimstra & Denissen, 2017; Rapee et al., 2019). Future studies should take more careful account of this.

## Conclusion

Social-emotional disorders are common during adolescence/emerging adulthood, yet few reviews synthesize existing research on the relationship between these disorders and ongoing identity development. This review identified 20 studies which examined longitudinal associations between identity development and social-emotional disorders in adolescents and emerging adults. The narrative synthesis found some evidence for bidirectional relationships between identity synthesis and confusion and socio-emotional disorders during adolescence and emerging adulthood. Meta-analyses of a sub-sample of these studies found no significant relationships between depression and anxiety symptoms and identity synthesis and confusion. This review highlights issues with the quality of research within the field, with all included studies only of fair or poor quality. There were shortfalls in selection and comparability of study groups (e.g., nonrepresentative samples; relevant factors not controlled for at baseline) and measurement of outcomes (e.g., self-report questionnaires; <70% retention rates). Further research should aim to address these shortfalls, so that clear conclusions might be drawn, and clinical recommendations made.

### Supplementary information

Supplementary Materials

## Appendix

Search Strategy

(Mental disorder OR mental illness OR mental health problem OR psychiatric disorder OR psychiatric disease OR anorexi* OR anxiety OR bipolar disorder OR bulim* OR depressi* OR eating disorder OR mania OR obsessive compulsive disorder OR OCD OR panic disorder OR personality disorder OR phobi* OR psychosis OR schizophrenia) *abstract, title* AND (identity development OR identity formation OR Erikson* OR identity style* OR identity process* OR identity confusion OR identity synthesis OR identity status* OR identity capital OR identity commitment OR identity exploration) *abstract, title*
