# Supplementary material for: Identity Development and Social-Emotional Disorders During Adolescence and Emerging Adulthood: A Systematic Review and Meta-Analysis
Source: J Youth Adolesc. 2021 Nov 16;51(1):16–29. doi: 10.1007/s10964-021-01536-7 (PMC8732894; doi:10.1007/s10964-021-01536-7)
Supplement: Supplementary file 1 — Supplementary Materials [file 10964_2021_1536_MOESM1_ESM.docx]

**Supplementary Materials**

**Online Resource 1**

*Characteristics and Results of Included studies*

| Cohort / Study | Sample | N | FU | Age | Measures | Quality | Main Findings | Meta-analyses |
| --- | --- | --- | --- | --- | --- | --- | --- | --- |
| CONAMORE (Dutch adolescents/ emerging adults) | | | | | | | |  |
| Crocetti et al., (2009) | *Early Adolescents*  49% female  Ethnicity NR  *Mid-Adolescents*  57% female  Ethnicity NR | 923  390 | 5 yrs. | 12.4 ± 0.6  10-15 yrs.  16.7 ± 0.8  16-20 yrs. | UMICS  SCARED | Fair | Adolescents in “high anxiety” and “low anxiety” trajectories did not differ in in-depth exploration. High anxiety trajectory associated with decreased identity commitment and increased reconsideration of commitment over time; low anxiety trajectory associated with increased identity commitment and decreased reconsideration of commitment over time. | 1, 2, 3 |
| Meeus et al., (2012) | *Early adolescents*  49% female  *Mid-adolescents*  57% female | 923  390 | 5 yrs. | 12.4 ± 0.6  10-15 yrs.  16.7 ± 0.8  16-20 yrs. | UMICS  CDI | Fair | *Total sample:* Moratorium and diffusion trajectories had greater depressive symptoms than achievers and early closure trajectories.  *Early adolescents:* Searching moratorium trajectory had fewer depressive symptoms than moratorium*.* No other significant differences. | Not included - duplicate |
| Schwartz et al., (2012) | 49% female  Ethnicity NR | 923 | 5 yrs. | 12.4 ± 0.6  10-15 yrs. | UMICS  SCARED  CDI | Poor | Identity commitment (but not reconsideration of commitment) predicted (-) depressive symptoms one year later.  Neither reconsideration of commitment nor identity commitment predicted anxiety disorder symptoms one year later.  Depressive symptoms predicted (-) identity commitment (but not reconsideration of commitment) one year later.  Anxiety symptoms predicted (+) reconsideration of commitment (but not identity commitment) one year later. | Not included - duplicate |
| Nelemans et al., (2014) | White  54% female | 239 | 8 yrs. | 12.7 ± 0.4  10-15 yrs. | UMICS  SCARED | Poor | Adolescents in “at-risk school anxiety” trajectory reported low, slightly decreasing levels of educational identity commitment from early adolescents into late adolescence**.** Adolescents in the “normal school anxiety” trajectory reported rapidly increasing educational identity commitment over time. No significant differences between “at-risk GAD” / “normal GAD” and “at-risk social anxiety” / “normal social anxiety” with regard to educational commitment. | Not included – No means / SDs |
| Van Doeselaar et al. (2018) #1 | *Early adolescents*  55% female  91% Dutch  *Mid-adolescents*  59% female  92% Dutch | 683  268 | 8 yrs. | 12.4 **±** 0.5  10-15 yrs.  16.6 **±** 0.8  16-20 yrs. | UMICS  CDI | Fair | Identity synthesis (career and interpersonal commitment) did not predict change in depressive symptoms over a four-year period in either age-group.  Depressive symptoms predicted (-) career identity commitment 4-5 years later, but not interpersonal identity commitment. | 1, 3 |
| COPAL (Hispanic Recent Migrants) | | | | | | | |  |
| Meca et al. (2019) | 47% female | 302 | 3 yrs. | 14.5 ± 0.9  14-17 yrs. | EPSI  CES-D | Fair | Within-person decreases in identity synthesis and increases in identity confusion predicted depressive symptoms six months later.  Within-person increases in depressive symptoms predicted identity confusion (+) synthesis (-) six months later. | 1, 2, 3 |
| Meca et al. (2017) | 47% female | 302 | 3 yrs. | 14.5 ± 0.9  14-17 yrs. | EPSI  CES-D | Poor | Identity synthesis did not predict depressive symptoms six months later. Identity confusion predicted (+) depressive symptoms six months later. | Not included – duplicate |
| Schwartz et al. (2017) | 47% female | 302 | 3 yrs. | 14.5 ± 0.9  14-17 yrs. | EPSI  CES-D | Fair | Identity confusion (but not identity synthesis) predicted (+) depressive symptoms six months later. | Not included – duplicate |
| JLIRP (Japanese adolescents) | | | | | | | |  |
| Hatano et al. (2019) | 50% female | 968 | 4 yrs. | NR  13-16 yrs. | UMICS  SDQ | Poor | *Educational identity:* Baseline depressive and anxiety disorder symptoms (but not change over time) associated with “achievement” (-) and “searching moratorium” (-) and “moratorium” (+) identity trajectories. Depressive and anxiety disorder symptoms not associated with “diffusion” and “diffusion to moratorium” trajectories.  *Interpersonal identity:* Baseline depressive and anxiety disorder symptoms (but not change over time) associated with achievement (-), moratorium (+), searching moratorium (-), and moratorium-diffusion-moratorium (+) identity trajectories. | 1, 2, 3 |
| Hatano et al. (2020) | 53% female  Ethnicity NR | 347 | 3 yrs. | 14 ± NR | DIDS  SDQ | Fair | Between-person change in ruminative exploration, commitment-making and exploration in breadth did not predict depressive symptoms. Within-person increase in ruminative exploration predicted (+) depressive symptoms one year later.  Between-person changes in depressive symptoms predicted (+) ruminative exploration one year later. Within-person changes in depressive symptoms did not predict identity synthesis (commitment-making; exploration in breadth) one year later. | 1, 2, 3 |
| LIA (Belgian adolescents / emerging adults) | | | | | | | |  |
| Verschueren et al., (2021) | 54% female  Ethnicity NR | 2612 | 2 yrs. | 14.6 ± 1.9  10-21 yrs. | EPSI  EDI-3 | Fair | Between-person change in identity synthesis (-) and identity confusion (+) predicted ED symptoms (drive for thinness, bulimia) one and two years later. | Not included – no depression / anxiety data |
| PALS (Belgian adolescents / emerging adults) | | | | | | | |  |
| Becht et al. (2019) #2 | 63% female  Ethnicity NR | 1022 | 4 yrs. | 15.8 ± 1.3  12-21 yrs. | DIDS  CES-D | Fair | Within-person increases in ruminative exploration predicted (+) depressive symptoms one year later. Other identity development variables did not predict depressive symptoms.  Within-person changes in depressive symptoms did not predict later identity development. | Not included – within-person focus |
| RADAR (Dutch adolescents) | | | | | | | |  |
| Becht et al. (2019) #1 | 43% female  Ethnicity NR | 497 | 5 yrs. | 14.0 ± 0.5  12-17 yrs. | U-MICS  RADS-2 | Fair | Within-person increase in reconsideration of commitment predicted (+) depressive symptoms one year later. Other identity development variables did not predict depressive symptoms.  Within-person changes in depressive symptoms did not predict later identity development. | Not included – within-person focus |
| Becht et al. (2016) | 43% female  Ethnicity NR | 494 | 5 yrs. | 13.0 ± 0.5  11-15 yrs. | UMICS SCARED | Fair | Adolescents in identity crisis trajectories had greater anxiety disorder symptoms than those in identity synthesis trajectories. Adolescents in educational identity crisis trajectory had greater school anxiety than those in educational identity synthesis trajectory. Adolescents in interpersonal crisis and synthesis trajectories did not differ with regard to school anxiety. | Not included – no means / SDs reported |
| Schwartz et al. (2011) | 45% female  Ethnicity NR | 580 | 1 yr. | 13.3 ± 0.5  11-15 yrs. | UMICS  RADS  SCARED | Poor | Fluctuations in reconsideration of commitment (but not mean level) predicted (+) depressive symptoms one year later.  Fluctuations in reconsideration of commitment (but not mean level) predicted (+) anxiety disorder symptoms one year later. | Not included – Within-person focus |
| USAD (Dutch adolescents) | | | | | | | | |
| Van Doeselaar et al. (2018) #2 | *Adolescents*  56% female  *Emerging Adults*  58% female | 960  944 | 3 yrs. | 15.0 **±** 1.7  12-17 yrs.  21.5 **±** 2.1  18-24 yrs. | UGIDS  GHQ | Fair | Interpersonal identity commitments predicted (-) depressive symptoms one and two years later (in both samples). Career identity commitment did not predict depressive symptoms.  Depressive symptoms did not predict interpersonal or career identity commitment one and two years later. | 1, 3 |
| Unspecified Cohort |  |  |  |  |  |  |  |  |
| Luyckx et al., (2013) | 85% female  uni students  94% Caucasian | 456 | 0.5 yrs. | 18.3 ± 1.4  17-29 yrs. | DIDS  ISRI  CES-D | Poor | Achievement and foreclosure trajectories had fewer depressive symptoms than all other trajectories. Troubled diffusion had greater depressive symptoms than all other trajectories. | 1, 2, 3 |
| Luyckx et al., (2008) | Female uni students  Ethnicity NR | 482 | 3 yrs. | 18.8 ± 0.6  Range NR | EIPQ  UMICS  CES-D | Poor | 21.6% of those in a “searching” identity trajectory (i.e., moratorium) were classified into an “optimal adjustment” trajectory, compared to 62.4% of participants in a “consolidating” identity trajectory (i.e., foreclosed) and 54.1% of those in a “path-making” identity trajectory (i.e., achieved). 33.0% of searchers were classified into a “stable maladjustment” trajectory, compared to 1.2% of the consolidators and 6.6% of the path-makers. | Not included – no means / SDs reported |
| Verschueren et al. (2018) | 51% female  Ethnicity NR | 530 | 3 yrs. | 15.0 ± 1.9  12-18 yrs. | EPSI  EDI-3 | Poor | Identity synthesis (-) and confusion (+) predicted ED symptoms one and two years later.  Body dissatisfaction and BN symptoms (but not drive for thinness) predicted identity synthesis (-) and confusion (+) one and two years later. | Not included – no depression / anxiety data |
| Verschueren et al. (2019) | 51% female  Ethnicity NR | 1528 | 3 yrs. | 15.0 ± 1.8  12-18 yrs. | EPSI  EDI-3  HADS | Poor | “Normal BMI / high symptoms” and “high BMI / high symptoms” trajectories had higher levels of identity confusion than “normal BMI / low symptoms” and “low BMI / low symptoms” trajectories. | 1, 2, 3 |

***Abbreviations:*** *BN = bulimia nervosa; CDI = Children’s Depression Inventory; CES-D = Centre for Epidemiologic Studies Depression Scale; DIDS = Dimensions of Identity Development Scale; ED = eating disorder; EDI-3 = Eating Disorder Inventory Version 3; EIPQ = Ego Identity Process Questionnaire; EPSI = Erikson Psychosocial Stage Inventory; GAD = generalised anxiety disorder; GHQ =General Health Questionnaire; HADS = Hamilton Anxiety and Depression Scale; ISRI = Identity Stage Resolution Index; NR = Not reported; RADS-2 = Reynolds Adolescent Depression Scale; SCARED = Screen for Child Anxiety Related Disorders; SD = Standard deviation; SDQ = Strengths and Difficulties Questionnaire; UGIDS = Utrecht Groningen Identity Development Scale; UMICS = Utrecht Management of Identity Commitments Scale.*

**Online Resource 2**

*Summary of Narrative Synthesis Results: Effect of Depression and Anxiety Symptoms on Identity Development*

| Study | IV | Significant Effect on DV (Identity) | | ES |
| --- | --- | --- | --- | --- |
|  |  | *Synthesis* | *Confusion* |  |
| Between-person |  |  |  |  |
| Hatano et al., (2020) | Dep / anx composite | No | Yes | 0.16** |
| Schwartz et al., (2012) | Dep / anx separately | Anx No  Dep Yes | Anx Yes  Dep No | 0.06*  - |
| Van Doesslar et al., (2018) #1 | Dep only | ED Yes  IP No | - | -0.06** |
| Van Doesslar et al., (2018) #2 | Dep / anx composite | No | -  - | -  - |
| Within-person |  |  |  |  |
| Becht et al., 2019 #1 | Dep | No | No | - |
| Becht et al., 2019 #2 | Dep | No | No | - |
| Hatano et al., 2020 | Dep / anx composite | No | No | - |
| Meca et al., 2019 | Dep | Yes | Yes | -0.16*, 0.28** |

Abbreviations: Anx = anxiety symptoms; DV = dependent variable; Dep = depressive symptoms; ED = educational; ES = effect size; IP = interpersonal; IV = independent variable

**Online Resource 3**

*Summary of Narrative Synthesis Results: Effect of Identity Development on Affective Disorder Symptoms*

| Study | IV | Significant Effect on DV (Affective disorder) | Effect Size |
| --- | --- | --- | --- |
| Between-person |  |  |  |
| Hatano et al., (2020) | Synthesis | No | - |
|  | Confusion | No | - |
| Meca et al., (2017) | Synthesis | No | - |
|  | Confusion | Yes | 0.26** |
| Schwartz et al., (2017) | Synthesis | No | - |
|  | Confusion | Yes | 0.40**** |
| Schwartz et al., (2012) | Synthesis | Anx No  Dep Yes | -  -0.08*** |
|  | Confusion | No | - |
| Schwartz et al., (2011) | Synthesis | No | - |
|  | Confusion | Yes | -0.40*** |
| Van Doesslar et al., (2018) #1 | Synthesis | No | - |
|  | Confusion | - | - |
| Van Doesslar et al., (2018) #2 | Synthesis | IP Yes  Ed No | -0.04*  - |
|  | Confusion | - | - |
| Within-person |  |  |  |
| Becht et al., (2019) #1 | Synthesis | No | - |
|  | Confusion | Yes | 0.10* |
| Becht et al., (2019) #2 | Synthesis | No | - |
|  | Confusion | Yes | 0.13* |
| Hatano et al., (2020) | Synthesis | No | - |
|  | Confusion | Yes | 0.26* |
| Meca et al., (2019) | Synthesis | Yes | -0.16* |
|  | Confusion | No | - |

**Abbreviations:** DV = dependent variable; ED = educational; ES = effect size; IP = interpersonal; IV = independent variable

**Online Resource 4**

*Data Extracted for Meta-Analyses*

|  |  | Identity Synthesis | | Identity Confusion | | Depression and Anxiety Symptoms | |
| --- | --- | --- | --- | --- | --- | --- | --- |
|  | Measures | Baseline  M (SD) | Follow-up  M (SD) | Baseline  M (SD) | Follow-up  M (SD) | Baseline  M (SD) | Follow-up  M (SD) |
| Crocetti et al., 2009 | UMICS  SCARED | 3.65 (0.69) | 3.75 (0.53) | 2.62 (1.61) | 2.54 (0.94) | 1.32 (0.27) | 1.25 (0.24) |
| Hatano et al., 2020 | DIDS  SDQ | 2.86 (0.93) | 2.95 (0.83) | 3.12 (0.73) | 3.14 (0.72) | 2.64 (0.66) | 2.66 (0.65) |
| Hatano et al., 2019 | UMICS  SDQ | 3.15 (0.68) | 3.24 (0.66) | 2.64 (0.81) | 2.95 (0.72) | 2.60 (0.55) | 2.59 (0.56) |
| Luyckx et al., 2013 | DIDS  CES-D | 3.39 (0.76) | 3.55 (0.76) | 3.35 (0.83) | 3.24 (0.86) | 0.92 (0.52) | 0.86 (0.50) |
| Meca et al., 2019 | EPSI  CES-D | 3.15 (0.57) | 2.98 (0.78) | 1.55 (0.76) | 1.52 (0.87) | 1.49 (0.78) | 1.44 (0.78) |
| Schwartz et al., 2012 | UMICS  SCARED  CDI | 3.65 (0.72) | 3.75 (0.57) | 2.03 (0.91 | 1.84 (0.73) | 1.24 (0.29) | 1.22 (0.23) |
| Van Doeselaar et al., 2018a | UMICS  CDI | 3.59 (0.77) | 3.72 (0.68) | - | - | 1.19 (0.27) | 1.13 (0.19) |
| Van Doeselaar et al., 2018b | UGIDS  GHQ | 3.55 (0.76) | 3.74 (0.68) | - | - | 1.36 (0.56) | 1.34 (0.55) |
| Verschueren et al., 2019 | EPSI  HADS | 3.71 (0.58) | 3.68 (0.65) | 2.61 (0.62) | 2.61 (0.66) | 0.79 (0.39) | 0.77 (0.44) |

***Abbreviations:*** *CDI = Children’s Depression Inventory; CES-D = Centre for Epidemiologic Studies Depression Scale; DIDS = Dimensions of Identity Development Scale; EPSI = Erikson Psychosocial Stage Inventory; GHQ =General Health Questionnaire; HADS = Hamilton Anxiety and Depression Scale; M = Mean; SCARED = Screen for Child Anxiety Related Disorders; SD = Standard deviation; SDQ = Strengths and Difficulties Questionnaire; UGIDS = Utrecht Groningen Identity Development Scale; U-MICS = Utrecht Management of Identity Commitments Scale.*
